# Why clinical training in China should improve: a cross-sectional study of MD graduates

**DOI:** 10.1186/s12909-021-02647-2

**Published:** 2021-05-10

**Authors:** Xiaoning Zhang, Chong Li, Cailing Yue, Xue Jiang, Junli Cao, Olle ten Cate

**Affiliations:** 1 School of Nursing, Xuzhou Medical University, Xuzhou, China; 2 Department of Neonatology, he Affiliated Hospital of Xuzhou Medical University, Xuzhou, China; 3 School of Nursing, Capital Medical University, Beijing, China; 4 Jiangsu Province Key Laboratory of Anesthesiology, Xuzhou Medical University, Xuzhou, China; 5 Jiangsu Province Key Laboratory of Anesthesia and Analgesia Application Technology, Xuzhou Medical University, Xuzhou, China; 6 Graduate School, Xuzhou Medical University, Xuzhou, China; 7 Center for Research and Development of Education, University Medical Center Utrecht, Utrecht, the Netherlands

**Keywords:** Doctor of medicine, Graduate, Clinical training

## Abstract

**Background:**

China is experiencing major medical education reforms that include establishing national training standards, standards for health professionals, and advanced health delivery system requirements. Graduate medical education (GME) is being piloted as a merger of Doctor of Medicine (MD) with PhD programs to improve academic research and clinical training. However, the academic degree-centred system has led to a preoccupation with research rather than clinical training. Unfortunately, there is a shortage of quality information regarding the clinical training of MD graduates from Chinese medical schools. To fill this gap, this general investigation aims to provide the perspective of recent MD graduates in China for the different subspecialties of clinical training as experienced in different contexts.

**Methods:**

There were 432 MD graduates who participated in an online survey regarding their clinical training. Information collected included overall satisfaction, educational supervision, supervised learning events, curriculum coverage, local teaching, teamwork, educational governance, workload, supportiveness of the environment, feedback, clinical experience, patient safety, handovers, and reporting systems.

**Results:**

Only 37.4% reported satisfaction with the overall clinical training quality; 54.6% rated the informal and bedside quality as “good”; 64.4% reported they knew who provided clinical supervision; but only 35.5% rated the quality of clinical supervision as high; 51.8% reported that they judged senior physicians as “not competent”; 41.9% agreed that the staff treated each other respectfully; 97.4% admitted that they worked beyond the mandatory hours and claimed they were regularly short of sleep; 84.2% raised concerns about patient safety; 45.3% reported that they received regular informal feedback; 48.1% believed that their concerns about education and training would be addressed.

**Conclusions:**

This study suggests that the quality of clinical training for MD graduates should be improved. While the overall satisfaction with the teaching quality was acceptable, the quality of many clinical training aspects scored poorly. A major problem seems an undue focus on research in MD/PhD training at the cost of the quality of clinical training, due to career perspectives that undervalue clinical competence. The findings of this study should benefit from a deeper investigation to understand the causes and possible remediation. Suggestions include defining subspecialties and training lengths; monitoring, evaluation, and integration SST with MD degree; providing funds or rewards for academic and clinical training; establishing supervising teams to guide clinical training; and establishing physician scientist task force to help overcome challenges.

**Supplementary Information:**

The online version contains supplementary material available at 10.1186/s12909-021-02647-2.

## Background

In China, no different from other countries across the globe, health professionals aim to deliver optimal clinical practice, but in practice the country has faced huge challenges to uphold its quality. In this rapidly developing country, clinical practice experiences a dual reform process, as both higher education and health care systems are under reform. The country is aware of a gap in medical education quality in comparison with several developed countries, and has launched reforms to bridge this with national quality standards, modern standards for health professionals, and advanced health delivery system requirements, thus meeting the health care demands of the Chinese public. In 2013, seven government ministries in China jointly launched a standard 8-year medical training model for the country, including an undergraduate phase and a standardised residency training (SRT) phase, together known as the” 5 + 3″ model, in a national strategy [1]. The first 5 y lead to a medical bachelor degree; the next 3 y to a master of medicine (MM) degree with SRT. China’s current medical workforce includes a vast range of practitioners, ranging from village ‘doctors’ with very limited education, many of whom practice traditional medicine [1], to doctors with a bachelor, master or doctorate degree, and to world class medical specialists with an advanced research and clinical training background. These highest level “physician-scientists” play important roles in medical education, research, and clinical practice in the large cities [2], combining clinical practice with academic research, and are committed to the quest for advanced knowledge and approaches to diagnose, treat, and prevent disease [3].

**Fig. 1:**
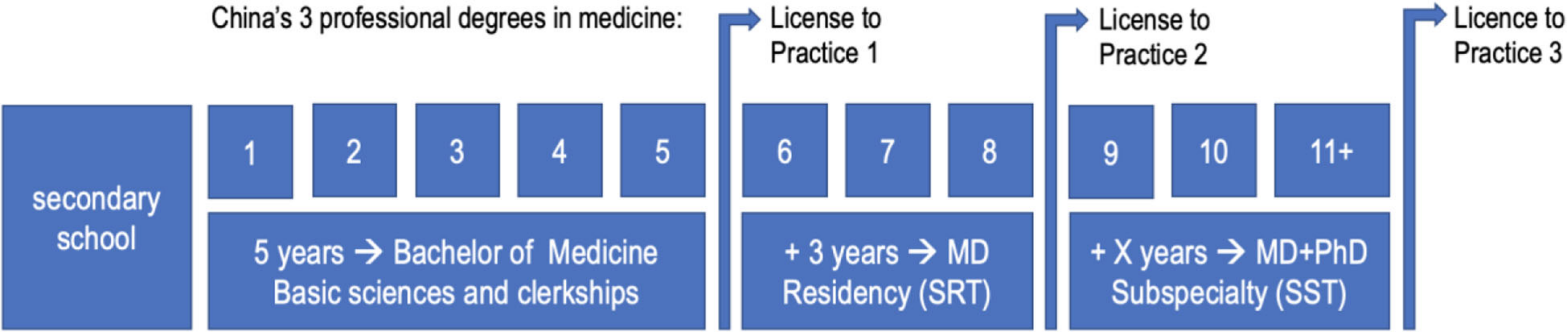
China’s new 5 + 3 + X model of medical education

Graduate medical education (GME) is now being piloted as a merger of MD and PhD programs at some high level universities to improve both academic research and clinical training. This training program provides integrated academic and clinical training for Doctor of Medicine (MD) graduates. China has built a degree and credentialing system, to some extent unique and to some extent comparable with developed countries, created during the rapidly changing start-up phase from 2013 to 2020 in parallel with the ambitious plan to improve physician quality. By the end of 2014 the National Health and Family Planning Commission (NHFPC, formerly the Ministry of Health) and the Ministry of Education (MoE) jointly reformed the medical professional degree program [1]. The full model now includes 5 y of undergraduate study (Bachelor of Medicine), followed by 3 y of SRT (leading to a Master of Medicine, MM degree), and an unspecified (X) number of years of standardised subspecialty training (SST) for Doctors of Medicine (MD degree), which phases are collectively referred to as the “5 + 3 + X” [undergraduate (5 y) + SRT (3 y) + SST (X y)] model (Fig. 1).

The third phase, the length of X is not specified, but China’s regulations do not allow for PhD trajectories longer than 8 years, the X-year MD + PhD program is controversial, particularly because of a highly ambitious combined clinical subspecialty and research training in “3+” y. In the US, the average training length of MD-PhD graduates increased from 6.5 y in 1980 to 8.0 y today [3]. MD-PhD programs at most US and a few Canadian medical schools incorporate 2 y of medical school, 4 y of the PhD degree, and another 2 y of medical school to complete the MD degree [3]. In addition, the aim of MD-PhD programs in medical education in several developed countries is to train highly qualified specialist practitioners, rather than to train those who focus only on academic research. Not everyone continues active research practices, but most continue active clinical practice.

The idealized graduate medical education (GME) system is more ambitious in China, as it combines clinical training and academic research with a physician-scientists career purpose. Back in 1956, Case Western Reserve University in the US was the first to launch an integrated MD-PhD program highlighting flexible and self-directed learning [4]. The difference between current models in China and the US is the integration of SRT and SST with the medical professional degree. By distinguishing professional degrees from academic degrees, the reformed GME requires the completion of a combination of SRT and MM degree; MM graduates obtain both an MM degree and certificate of SRT (MM degree with SRT), while MD graduates must undergo SST (MD degree with SST) [1]. The MD-PhD (referred to as an MD program in this study) is the highest level of medical education, and is a critical pathway to the training of next-generation physician-scientists in China [5]. This idealized career pathway, leading to the higher salaries and reputation, has however been criticized recently for concentrating primarily on academic research, while leaving clinical practice training inadequate [6]. This academic degree-centred system has led to a preoccupation with academic research, rather than clinical training in China, and the system is criticized for inadequate subspecialist clinical proficiency.

In developed countries physician-scientists play crucial roles in medical research and education and care [7], and have enhanced skills by integrating MD-PhD training in the context of dual-degree programs [4]. In China, although clinical training is a core competency required to be a physician-scientist, the goal of the MD-PhD phase is primarily to train scientific research and academic competence [8]. Concerns have been raised about the clinical proficiency of these MD graduates, having deficits in clinical practice experience as compared with peer physicians who do not pursue the “3+ X”-phase [9].

To date, comprehensive quality data regarding the clinical training of MD graduates are not available in China. This study aimed to fill this gap, by investigating the current clinical training of MD graduates, to provide crucial evidence to examine and improve MD training quality, and to explore developmental trends in GME to contribute to policy considerations underpinning high quality GME in China. This study may help identify areas of strengths and areas that fail to meet acceptable standards.

## Methods

### Respondents

This study was a cross-sectional design, in which a self-report questionnaire was used to evaluate the clinical training of MD graduates. The inclusion criteria focused on respondents studying at different SST stages, interested to participate. Purposive sampling was conducted [10] and based on a pilot study, the intended sample size was about 350. An attempt was made to achieve as much diversity as possible in the research population across medical schools. Questionnaires requesting participation were sent to the administrators at a national medical education conference. MD graduates were defined as those studying at the time that this study was conducted; 432 MD graduates from informal academic organisations in 22 universities agreed to participate from 1 January to 1 March 2017 via an online survey. As compensation, respondents received an incentive worth ¥10.00 on the website.

### Data collection

The questionnaire was modified from the General Medical Council (GMC) National Trainee Survey [11], monitoring the quality of clinical training and education of doctors in the UK [12], in an effort to evaluate the quality of postgraduate medical education and doctors in clinical training every year using international comparative standards. Participants in this study were MD graduates, most of them with a Chinese medical license and the questionnaire was deemed optimal for this group based on a literature review. The questionnaire included questions on basic information, “overall satisfaction,” “adequate experience,” “workload,” “clinical supervision,” and “educational supervision”. Two bilingual researchers translated the initial questionnaire from English to Chinese using the back-translation technique based on a literature review [13]. Discrepancies between the original English and back-translated Chinese versions were discussed among the bilingual researchers until they reached a consensus regarding the linguistic and cultural equivalences.

The questionnaire was pretested on 10 respondents in advance to ensure that it was easily understood before undertaking the pilot study. A pilot study for the questionnaire was conducted to improve its clarity, consistency, and validity; it included the participation of 15 respondents who were representative of the target population, and the questionnaire was not changed after the pilot study. The questionnaire was modified and validated by a panel of nine expert members, including experts working in different Chinese GME subspecialties and MD supervisors who were interested in the study and willing to participate.

The 11 components of the final questionnaire focused primarily on clinical training, and included demographic data (sex, age, working time, professional title, living situation), overall satisfaction, educational supervision, supervised learning events, curriculum coverage, local teaching, teamwork, educational governance, workload, supportiveness of the environment, feedback, clinical experience, patient safety, handover, and reporting systems. Response formats included multiple choices with three to six options, yes/no responses, Likert-type scales, and open-ended questions.

To improve responses and obtain the target number of respondents, they completed the questionnaire via computers or smart devices, scanned QR code by social media app. If respondents attempted to move on to the next question without answering the current question, a warning appeared and they were directed back to the unanswered question. The IP addresses and social media accounts were used to identify and enable eliminating duplicate participant responses. Responses were examined for indications of systematic response bias (e.g., clicking the same response option to move rapidly through the questionnaire).

Potential respondents received a summary of the study proposal detailing the research aim, procedure, expected outcomes, risks, benefits, and their rights to withdraw. Respondents were recruited on social media app; the first page of the questionnaire provided an outline and guide to the study, and ethical rules were followed with regards to data protection and the storage of personal data; all information collected was anonymous [14]. This study received ethical approval by the Institutional Review Board of Xuzhou Medical University (Number: 201568), and informed consent was provided after the study guide. All respondents signed their names on the online informed consent form.

### Statistical analysis

The data were analysed using Microsoft Excel 2016 (Microsoft Corporation, Redmond, Washington, USA) for Windows. Descriptive analysis (means, standard deviations, and percentages) was used to quantify the responses and summarise the variables.

## Results

There were 480 respondents who were invited to participate in the study and 432 responded (response rate 90.0%), from 22 universities and 20 cities. The respondents’ mean age was 28 ± 3.6 y; 54.4% were married, 64.0% were men, 24.9% stated they never had a job; 69.5% had obtained a professional degree of master of medicine (MM), and 88.9% had obtained a SRT certificate. The findings were presented in tables (supplemental materials) and figures.

Of the respondents, 45.4% reported they received formative feedback from senior physicians at least once a month, while 22.6% responded they had never received formative feedback. Moreover, 51.8% reported they had received feedback about their progress from their educational supervisors, but 37.7% did not consider the feedback received to be useful, and only 32.6% reported they had received useful formal assessments of performance in the workplace. Additionally, 61.2% had agreed on educational objectives with their educational supervisors, and 64.4% indicated they had a training agreement with their educational supervisors set their respective responsibilities. While 84.2% had raised concerns about patient safety, 74.4% reported their concerns had been resolved or were being addressed.

**Fig. 2:**
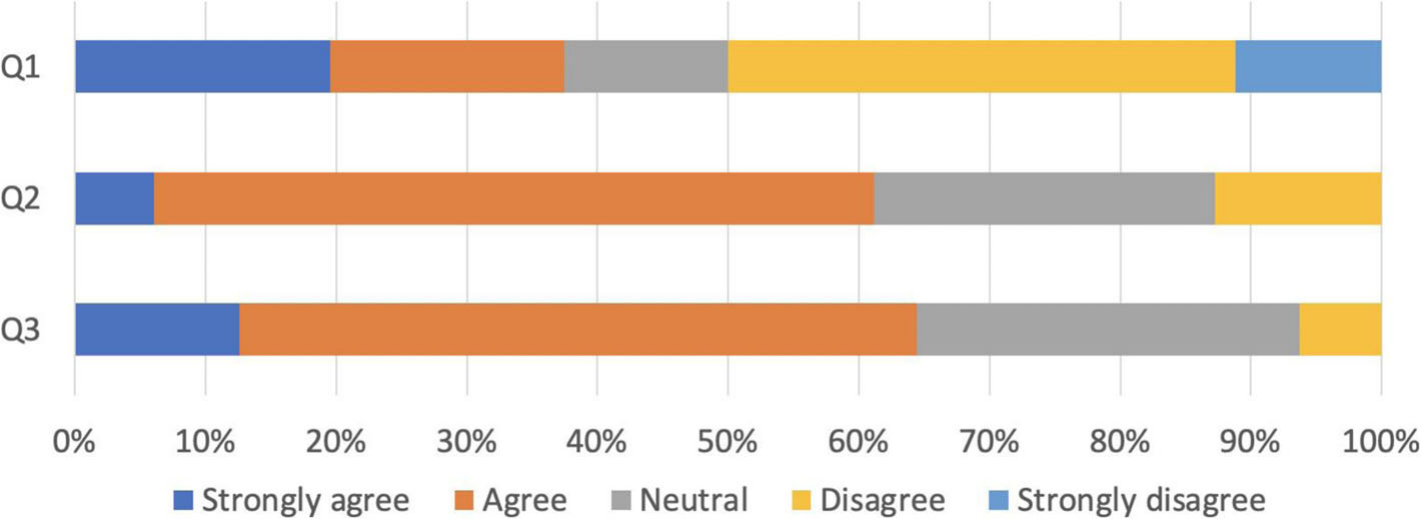
Satisfaction with training and confidence about career (*N* = 430). Q1: “I am satisfied with the quality of training in clinical practice” . O2: “I am confident that MD career will help me acquire the competencies I need at my current stage of training”. O3: “This post will be useful for my future career”

Figure 2 shows that 61.2% of the respondents believed an MD career would ensure that they acquired the competencies they needed, and these would be useful for their future career, but only a third agreed with the statement of satisfaction with the overall quality of clinical training.

**Fig. 3:**
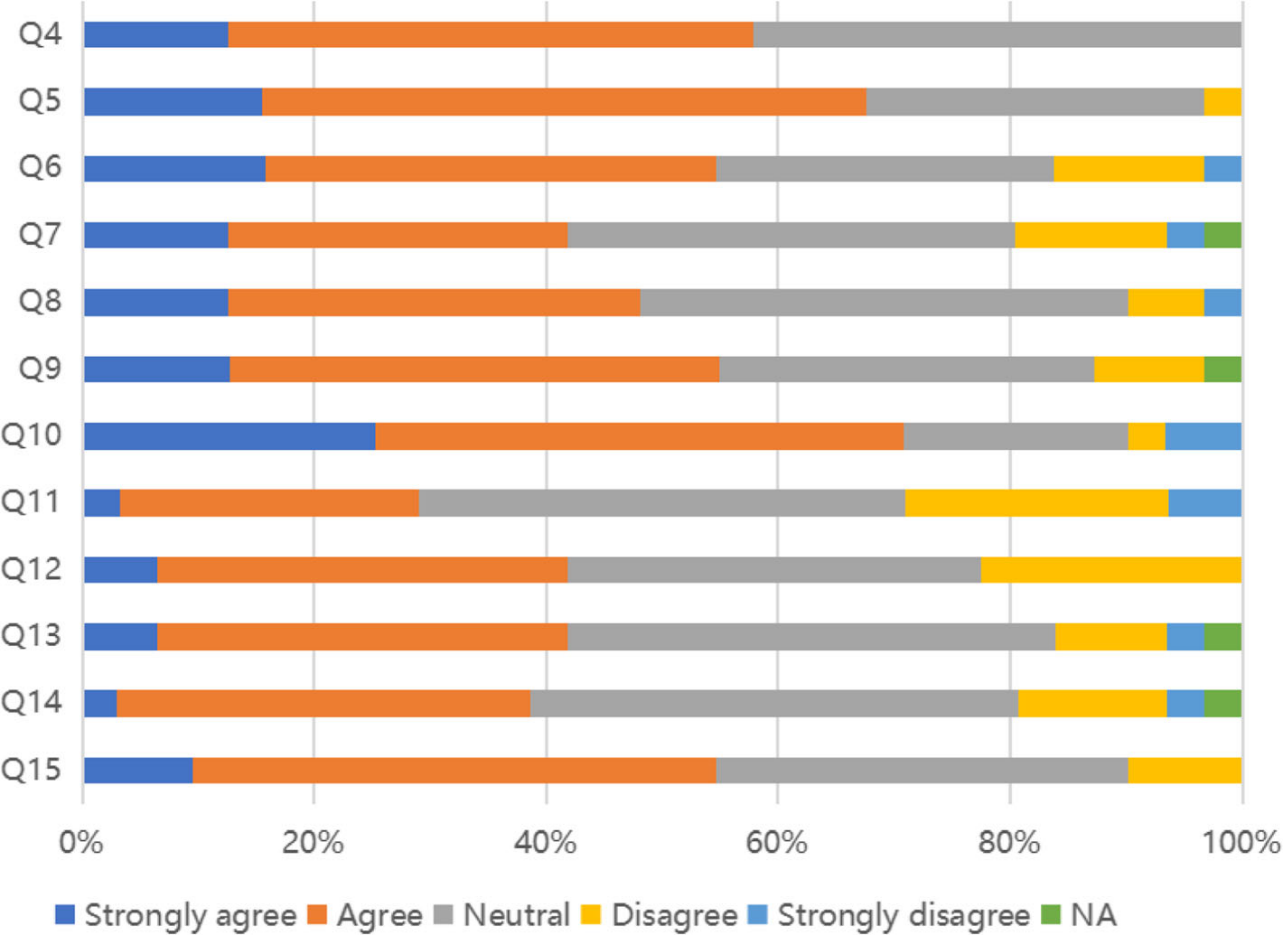
Satisfaction with the educational climate of the program (*N* = 430). Q4: My organization encourages teamwork culture between multidiscipline healthcare professionals. Q5: My organization encourages teamwork culture between clinical departments. Q6: If I asked for help from outside my department, I’m confident I would receive it. Q7: I am confident that I know how, or could find out how, to raise a concern about my education and training. Q8: If I were to raise a concern about my education and training, I’m confident it would be addressed. Q9: I am confident that I know how, or could find out how, to escalate such a concern if I felt it wasn’t being addressed. Q10: The training environment is fully supportive. Q11: Staff is always treated fairly. Q12: Staff always treats each other with respect. Q13: The training environment is one that fully supports the confidence building of physicians in training. Q14: If I were to disagree with senior physicians, they would be open to my opinion. Q15: If I had any concerns (personal or educational) I would know who to approach to talk to in confidence

Figure 3 shows that interprofessional and multidisciplinary collaborations are generally encouraged and present, 70.8% of the respondents agreed or strongly agreed that in a supportive culture that allows for speaking up in case of concerns. The respondents were less positive about the fair treatment of clinical supervisors, 29.1% agreed or strongly agreed that staff was always treated fairly, but also about their receptiveness to direct feedback from them.

**Fig. 4:**
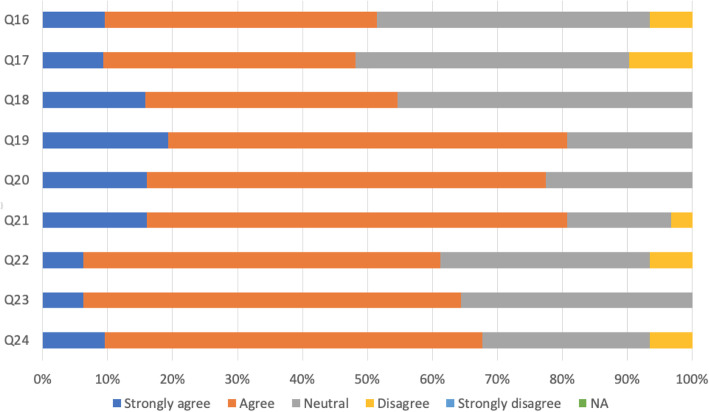
Curriculum coverage and supervised learning events (*N* = 430). Q16: I’m confident that this post will give the opportunities to meet cultivation objectives in: professional experience (leadership, teaching, research, and quality improvement etc.). Q17: I’m confident that this post will give the opportunities to meet cultivation objectives in: practical experience (procedures and treatments of chest drains, passing NG tubes, minor surgeries under local anesthetic, biopsies, fitting coils, injections, psychological therapies etc.). Q18: I’m confident that this post will give the opportunities to meet training objectives in: clinical experience (examination skills, taking a history, deciding investigations and management, seeing a variety of patients in different settings etc.). Q19: Supervised learning events (SLEs) have led to me reflecting on my clinical practice. Q20: SLEs have helped me to identify areas in which I need to develop. Q21: SLEs have enabled me to improve my practice. Q22: How easy or difficult was it to get a suitable physician to complete an SLE with you?. Q23: I have access to a senior physician who is onsite at all times. Q24: The senior physician onsite could advise on any clinical situation

As shown in Fig. 4, a large majority of respondents agreed supervised learning events (SLEs) made them reflect on clinical practice, had helped them to identify and develop clinical practice gaps and enabled them to improve clinical practice. Most agreed it was easy to obtain SLEs from proper physicians, contact onsite senior physicians at all times and receive their advice on any clinical situation. About half were confident the curriculum would meet their objectives related to professional experience (leadership, teaching, research, and quality improvement) and that an MD career would meet their objectives for clinical practical experience for procedures and treatments.

**Fig. 5:**
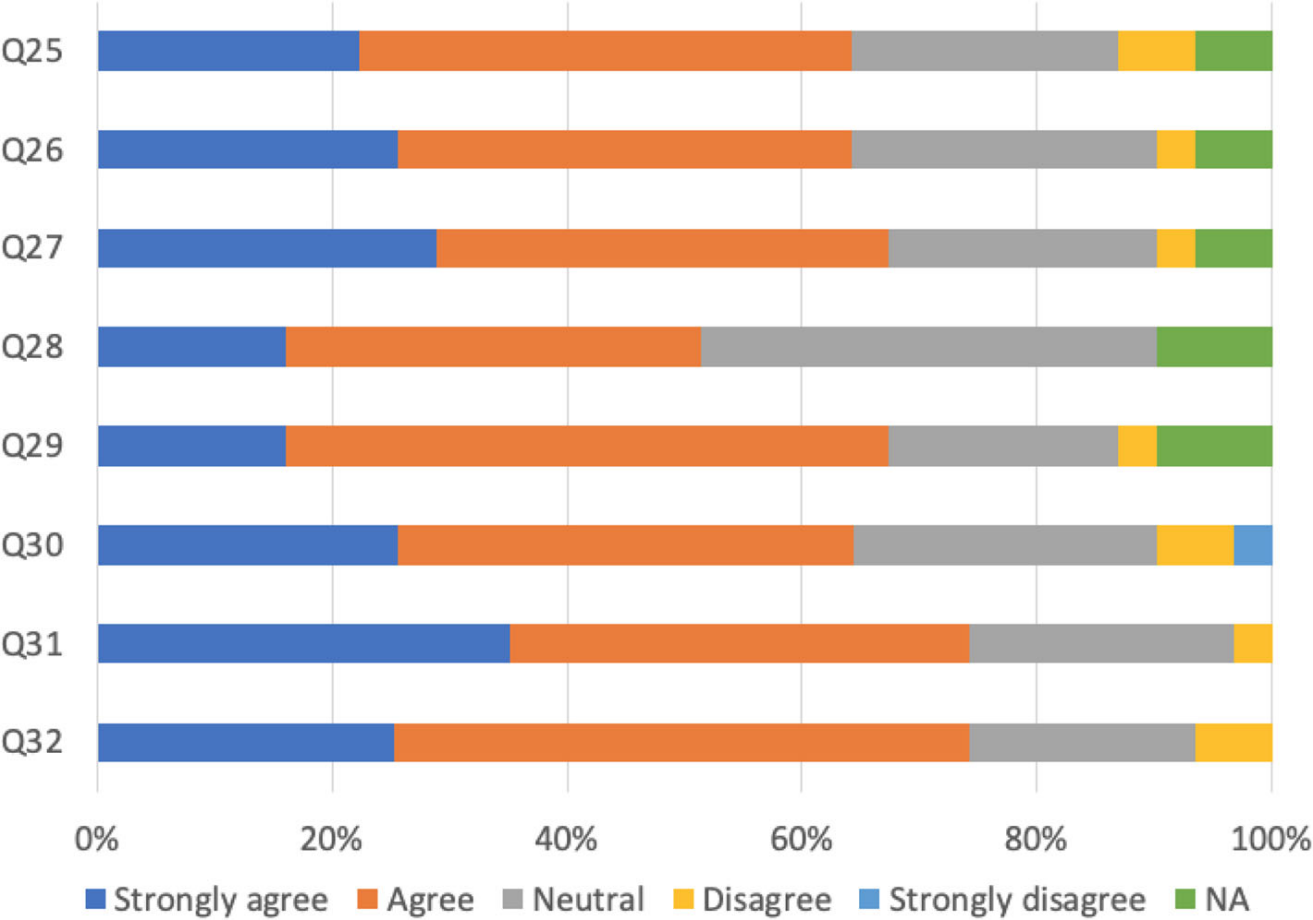
Satisfaction the culture of patient safety (*N* = 430). Q25: Handover arrangements always ensure continuity of care for patients between shifts. Q26: Handover arrangements always ensure continuity of care for patients between departments. Q27: Appropriate members of the multidisciplinary team are included in handover. Q28: I have been made aware of how to report patient safety incidents and near misses. Q29: There is a culture of proactively reporting concerns. Q30: There is a culture of learning lessons from concerns raised. Q31: I am confident that concerns are effectively dealt with. Q32: When concerns are raised, the subsequent actions are fed back appropriately

The majority of respondents showed satisfaction (Fig. 5) with the culture of patient safety and patient handovers between shifts, across departments and disciplines, including how to report incidents, near-misses and concerns. Of 64.2% of the respondents were confident that concerns were effectively dealt with when they were raised and fed back appropriately.

**Fig. 6:**
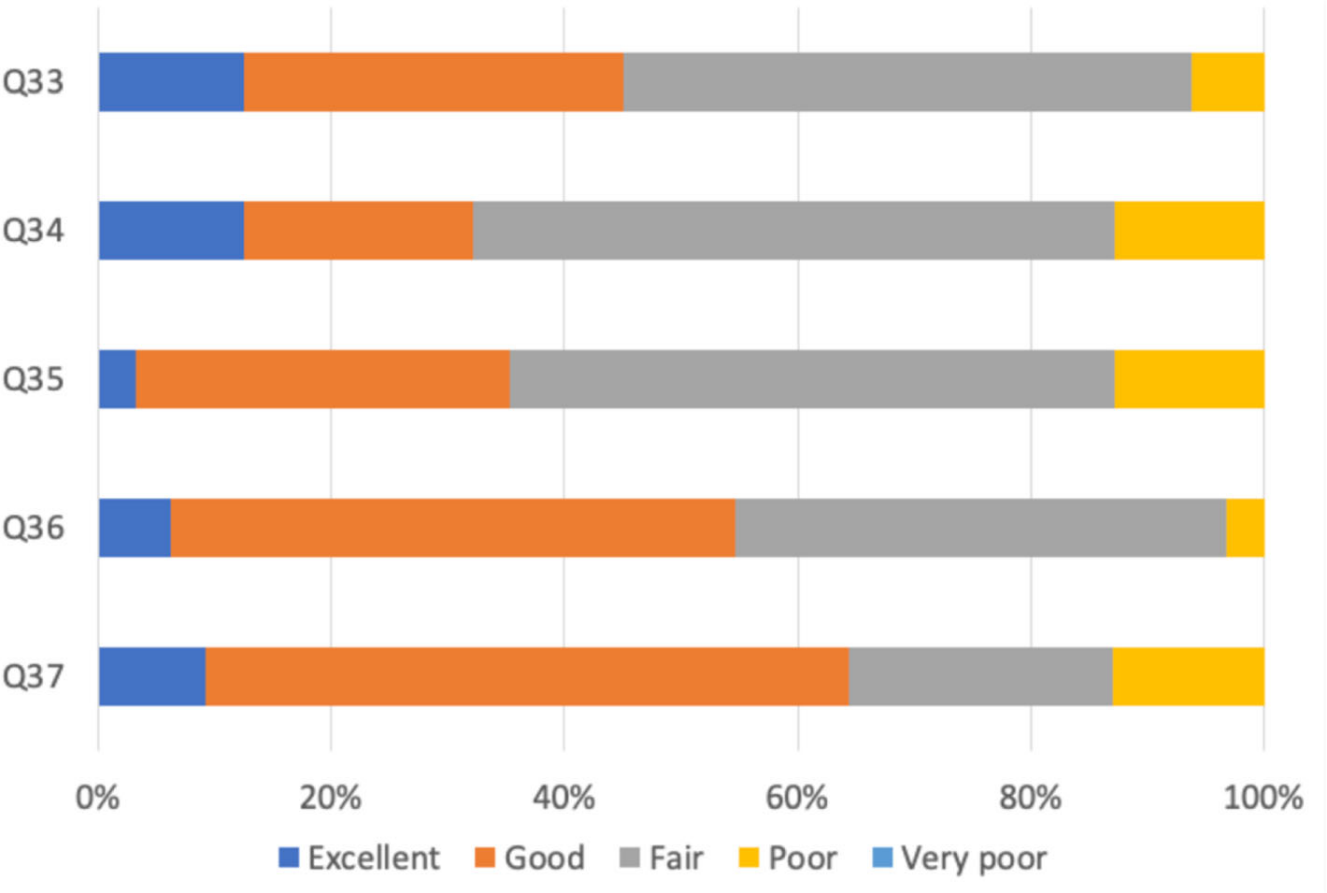
Quality of teaching, supervision and experience (*N* = 430. Q33: How would you rate the quality of the local/departmental teaching?. Q34: How would you rate the quality of teaching (informal and bedside teaching as well as formal and organized sessions)?. Q35: How would you rate the quality of clinical supervision?. Q36: How would you rate the quality of clinical experience?. Q37: How would you rate the practical experience you were receiving?

As Fig. 6 shows, 64.4% of the respondents was satisfied with the teaching received, but only a minority with the clinical supervision and experience.

**Fig. 7:**
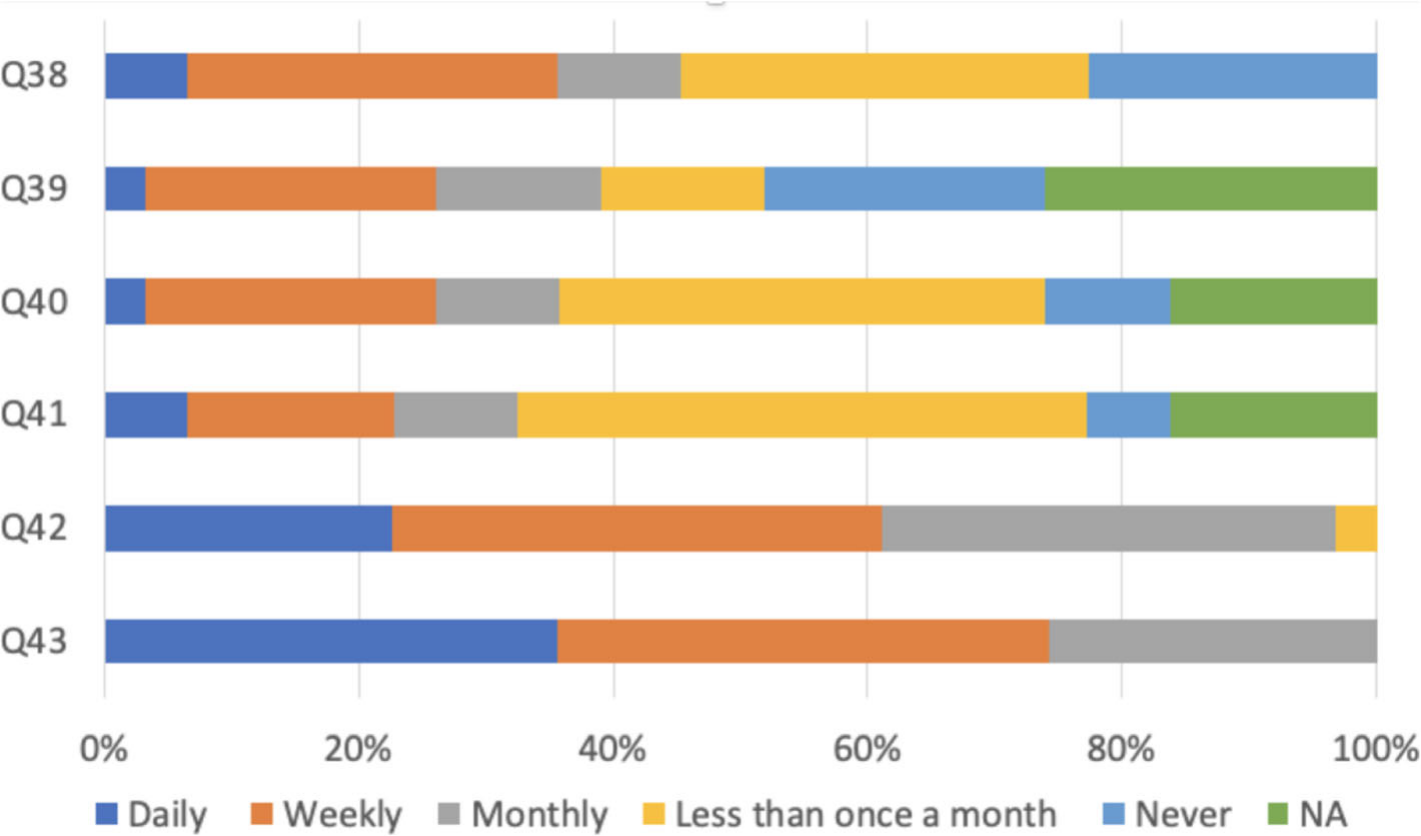
Feedback, clinical supervision and workload (*N* = 430). Q38: How often (if at all) do you receive informal feedback from senior physicians about your performance?. Q39: How often (if ever) are you supervised by someone who you feel isn’t competent to do so?. Q40: How often (if ever) do you feel forced to cope with clinical problems beyond your competence or experience?. Q41: How often (if ever) are you expected to obtain consent for procedures when you feel you do not understand the proposed intervention and its risks?. Q42: Have you worked out of hours (this includes night shifts and weekends)?. Q43: How often (if at all) does your working pattern leave you feeling short of sleep when at work?

As Fig. 7 shows, many respondents reported being often supervised by senior physicians they felt incompetent to do, and forced to frequently cope with clinical problems beyond their competence or experience, or were expected to obtain consent for procedures for which they did not understand the risks of the proposed intervention. All respondents admitted that they worked out of hours, including night shifts and weekends and all claimed to some extent short on sleep while at work in their current working pattern.

## Discussion

To the best of the authors’ knowledge, this is the first analysis to explore the quality of the clinical training of Chinese MD graduates from a range of subspecialties and at different SST stages. MD graduates do not receive the optimal combination of clinical training and academic research in China. This study indicates that the participating MD graduates were a young group with primary degrees, and most had experienced short-term clinical training before entering the MD program. The findings suggest that the overall satisfaction, the training environment, feedback, clinical experiences, clinical teaching, and supervision should be improved.

This study identified some challenges in clinical training that should be addressed. The clinical competence of Chinese physicians, even those with higher academic (PhD) degrees, is often judged only by academic research and output, not by their clinical teaching competence. MD graduates face higher demands of academic research than of clinical training. They are generally unable to graduate if they have not published in one or more English journals with a high impact factor. In addition, MD-PhD graduates must have completed more academic research, at the cost of essential clinical training time [2], there is no balance in the combination of clinical training and academic research [15]. In this study, MD graduates stated that they needed more support to balance clinical training and academic research, which is consistent with the situation of MD-PhD programs in the US. Though academic research training has been neglected in the US for many years [6, 16]; another study reported that graduates from 24 MD-PhD programs spent 75 to 80% of their time conducting academic research [3].

Most MD graduates who participated in this study had previous job experience, which is different from most MD-PhD students in the US [3]; however, professionalism in patient care is reported to be substandard and requires improvement [17, 18]. The inadequate instructional abilities and professionalism of preceptors were commented on frequently by our respondents [2]. The present study revealed that the clinical training of MD graduates, especially supervision and feedback, should be improved. Poor levels of clinical supervision create an unsafe and unsupportive clinical environment, and improper clinical supervision may impact patient safety and health outcomes. Although MD graduates expressed satisfaction with the quality of preceptors, it seemed that the preceptors provided them with adequate training. This study indicates the importance of highlighting clinical supervision and raises concerns about frequency and effect of feedback. Clinical training environments present challenges including patient care and multidisciplinary pressures on clinical training and supervision [19]. This pressure exists in health care services across China and preceptors are experiencing similar pressures, thus raising concerns about the overall balance between clinical training received and routine clinical work expected to be performed [20].

There is a need to protect, enhance, and recognise the importance of preceptors, and to provide consistent ongoing support to them. MD graduates and preceptors are working together to improve health care services, and it is clear that medical education is a priority for the country. Health care services provision and medical education are inextricably linked, and a lack of training opportunities and a busy working environment influence patient safety and care. Another important area to explore is the health and wellbeing of MD graduates, and the growing concerns about the impact of the working environment on individuals.

Physicians with higher-level degrees (i.e. doctorate degrees) are more likely to find higher-paying jobs in higher-level hospitals and larger cities, where they could also obtain high-quality and organised clinical training associated with medical career progression and increased future income. Very few MD physicians serve health care in rural areas [3]. A considerable proportion of Shanghai SRT trainees terminated this training to enter an MD-PhD program; this doctorate degree being more attractive for their careers than an SRT certificate [1]. Without a valid 3-y MM with SRT, graduates cannot enter an MD program. MD graduates normally complete an examination organised by universities and have a curriculum vitae and expert recommendation letters. After passing the examination, candidates are interviewed by an expert panel, including supervisors. The admission for MD program is unimaginable without an entrance examination, in contrast to the previous strict national entrance examination. These elite positions are extremely competitive. In the USA, a Medical College Admission Test (MCAT) is valid for admission in all medical schools [21]. A similar admission test should be considered in China for entrance into a MD program to ensure fairness.

### Implications for future MD program developments in China

How can training quality in the combination of an MD degree and SST be ensured? Current complex clinical environments challenge the viability of China’s contemporary clinical training for emergency patient care, using advanced technology, and for multidisciplinary cooperation for mentoring [19]. The accreditation of SRT and SST training institutions is essential and the Chinese Medical Doctor Association (CMDA) has been designated to manage accreditation; within a very short period, it has accredited approximately 500 training hospitals spread geographically [1]. The validity of accreditation decisions may be questioned because of a lack of professional expertise, acceptance of its authority, and financial resources. The standards of SRT and SST may not provide trainees with the clinical competencies MD graduates require. High-quality professional expertise is only present in a few top hospitals; therefore, advanced certification mechanism needs to be established [1]. Moreover, with the support of the China Medical Board (CMB), 24 training hospitals have recently developed the China Consortium of Elite Medical Schools. Medical education reform may depend upon the capacity of Chinese professional associations in the future.

MD-PhD programs in the US and institutional, federal, and societal programs provide full tuition and a stipend to support graduates’ training [3], MD graduates obtain funding to support laboratory-based research [3]. In contrast with USA, the funding of academic research training in China is very limited. The MD-PhD trajectories are therefore short, but do demand research output, which consequently leads to compromised clinical experience and training. Knowing the clinical training status can help predict and prevent training problems at an earlier stage. More effective recommendations in this critical area should be actively considered. All training hospitals should depend on local conditions, have the approval and the capacity to support the clinical training, which can help develop adequate competencies and maintain optimal clinical training. Policymakers should reflect current clinical training to provide and refine sustainable guidance to support MD preceptors to make improvements where necessary, and consider more flexible clinical training programs. The training standards should outline how MD graduates can be treated more professionally at all SST stages and individuals clinical training components [6]. Policymakers should cooperate with stakeholders before the quality deteriorates and causes harm to patient care and undermines clinical training and physicians’ motivation [22]. The need to further improve the MD program as being broadly felt, and is supported by the results of this survey [23].

The recommendations include (a) defining subspecialties and their respective training lengths and pathways based on existing SRT; the monitoring, evaluation, and integration of SST with an MD degree must be developed systematically and sustainably; (b) providing targeted individual-dominated funds or rewards for academic and clinical training, (c) establishing supervising teams to guide clinical training; and finally (d) a physician scientist task force may be established to help overcome challenges at various training stages [24], especially funding, individual training, supervision, and feedback [25].

Overall, MD graduates in China do not receive the optimal combination of clinical and academic research training. Supervision of, and feedback on, clinical training for MD graduates can be improved. Only a few top hospitals present high-quality professional expertise, and advanced certification mechanism needs to be established. Policymakers should pay close attention to those institutions that do not deliver on all responsibilities for the training of MD graduates belong to the training of MD graduates. Preceptors should be selected, inducted, trained, and appraised to reflect clinical training. Suggestions include improving safe and effective care, providing positive clinical supervision, offering appropriate practice opportunities, providing health care services, and maintaining optimal patient safety in challenging times. Current clinical training should provide refined and sustainable guidance to make improvements where necessary.

### Strengths and limitations

This cross-sectional study based on purposive sampling and self-reporting, was limited by a relatively small sample size, the subspecialties of respondents weren’t collected accurately, which did not combine items into indicators, and therefore cannot be generalised to all Chinese MD graduates. The strengths of this study include its investigation of clinical training independently from academic research. In subsequent study, optimal integrated clinical training and academic aspects should be developed. The respondents may have provided socially desirable answers to the questionnaire due to the Chinese culture, as many of the answers were not very negative. All comments were collected from open-ended questions, and interviews were not conducted, which would be useful in future studies to provide background information. A national cohort study is needed, and it is suggested that in-depth and national clinical training cohort studies be conducted yearly. This study was, because of a relatively small population, not suitable for factor analysis, in pursuit of underlying factors, not for identifying the relationships between variables and the best predictor of overall satisfaction. Future studies, more systematically and among a larger population may be investigated or examined to reveal such factors, as well as linking such data to characteristics of training hospitals, combining items into indicators.

## Conclusions

With the combination of an MD degree and SST certificate in this round of GME reform, Chinese MD graduates do not receive the optimal combination of clinical and academic research training. This study presents an overview of the clinical training of MD programs, identifies poor training standards of MD graduates, highlights that the expertise of preceptors should be elevated, and reveals that clinical training should be improved. Most surveyed MD graduates reported satisfaction with the clinical training they received, but the quality of the overall satisfaction, training environment, feedback, clinical teaching, and supervision should be improved. Each of these aspects may enable a deeper understanding of causes and possible remediation. A supportive training environment should be created and strategies should be developed to balance academic research and clinical training to ensure that both MD graduates and preceptors receive the best possible support.

## Supplementary Information

**Additional file 1: **Supplemental Materials With MEED-D-19-00500R4. **Table 1** Overall satisfaction, adequate experience, teamwork, educational governance, supportive environment, curriculum coverage, supervised learning events, handover and reporting systems (*N*=430). **Table 2** Clinical experience, clinical supervision, local teaching and overall satisfaction (*N*=430). **Table 3** Feedback frequency, clinical supervision and workload (*N*=430).

## Data Availability

The datasets that support the findings of this study are available from the corresponding author upon reasonable request.

## Abbreviations

CMB: China Medical Board
CMDA: Chinese Medical Doctor Association
GMC: General Medical Council
GME: Graduate Medical Education
MCAT: Medical College Admission Test
MD: Doctor of Medicine
MM: Master of Medicine
MoE: Ministry of Education
MSTP: Medical Scientist Training Program
NHFPC: National Health and Family Planning Commission, formerly the Ministry of Health
SCOPSR: State Commission Office for Public Sector Reform
SLEs: Supervised learning events
SRT: Standardised residency training
SST: Standardised subspecialty training
