# Supplementary material for: Why clinical training in China should improve: a cross-sectional study of MD graduates
Source: BMC Med Educ. 2021 May 10;21:266. doi: 10.1186/s12909-021-02647-2 (PMC8108351; doi:10.1186/s12909-021-02647-2)
Supplement: Supplementary file 1 — Additional file 1: Supplemental Materials With MEED-D-19-00500R4. Table 1 Overall satisfaction, adequate experience, teamwork, educational governance, supportive environment, curriculum coverage, supervised learning events, handover and reporting systems (N=430). Table 2 Clinical experience, clinical supervision, local teaching and overall satisfaction (N=430). Table 3 Feedback frequency, clinical supervision and workload (N=430). [file 12909_2021_2647_MOESM1_ESM.docx]

**SUPPLEMENTAL MATERIALS WITH MEED-D-19-00500R4**

**Table 1 Overall satisfaction, adequate experience, teamwork, educational governance, supportive environment, curriculum coverage, supervised learning events, handover and reporting systems (n=430)**

| Overall satisfaction and adequate experience | Strongly agree, n (%) | Agree, n (%) | Neutral, n (%) | Disagree, n (%) | Strongly disagree, n (%) |  |
| --- | --- | --- | --- | --- | --- | --- |
| I am satisfied with the quality of training in clinical practice. | 84（19.53） | 77（17.91） | 54（12.56） | 167（38.84） | 48（11.16） |  |
| I am confident that MD career will help me acquire the competencies I need at my current stage of training. | 26（6.04） | 237（55.12） | 112（26.05） | 55（12.79） | 0（0.00） |  |
| This post will be useful for my future career. | 54（12.56） | 223（51.86） | 126（29.30） | 27（6.28） | 0（0.00） |  |
| Teamwork, educational governance and supportive environment | Strongly agree, n (%) | Agree, n (%) | Neutral, n (%) | Disagree, n (%) | Strongly disagree, n (%) | NA, n (%) |
| My organization encourages teamwork culture between multidiscipline healthcare professionals. | 54（12.56） | 195（45.35） | 181（42.09） | 0（0.00） | 0（0.00） | 0（0.00） |
| My organization encourages teamwork culture between clinical departments. | 67（15.58） | 224（52.09） | 125（29.30） | 14（3.26） | 0（0.00） | 0（0.00） |
| If I asked for help from outside my department, I’m confident I would receive it. | 68（15.81） | 167（38.84） | 125（29.07） | 56（13.02） | 14（3.26） | 0（0.00） |
| I am confident that I know how, or could find out how, to raise a concern about my education and training. | 54（12.56） | 126（29.30） | 166（38.60） | 56（13.02） | 14（3.26） | 14（3.26） |
| If I were to raise a concern about my education and training, I’m confident it would be addressed. | 54（12.56） | 153（35.58） | 181（42.09） | 28（6.51） | 14（3.26） | 11（2.56） |
| I am confident that I know how, or could find out how, to escalate such a concern if I felt it wasn’t being addressed. | 55（12.79） | 181（42.09） | 139（32.33） | 41（9.53） | 0（0.00） | 14（2.69） |
| The training environment is fully supportive. | 109（25.35） | 196（45.43） | 83（19.30） | 14（3.26） | 28（6.51） | 0（0.00） |
| Staff is always treated fairly. | 14（3.26） | 111（25.81） | 180（41.86） | 98（22.79） | 27（6.28） | 0（0.00） |
| Staff always treats each other with respect. | 28（6.51） | 152（35.35） | 153（35.58） | 97（22.56） | 0（0.00） | 0（0.00） |
| The training environment is one that fully supports the confidence building of physicians in training. | 28（6.51） | 152（35.35） | 181（42.09） | 41（9.53） | 14（3.26） | 14（3.26） |
| If I were to disagree with senior physicians, they would be open to my opinion. | 13（3.02） | 153（35.58） | 181（42.09） | 55（12.79） | 14（3.26） | 14（3.26） |
| If I had any concerns (personal or educational) I would know who to approach to talk to in confidence. | 41（9.53） | 194（45.12） | 153（35.58） | 42（9.77） | 0（0.00） | 0（0.00） |
| Curriculum coverage and supervised learning events | | | | | | |
| I'm confident that this post will give the opportunities to meet cultivation objectives in: PROFESSIONAL EXPERIENCE (leadership, teaching, research, and quality improvement etc.) | 41（9.53） | 180（41.86） | 181（42.09） | 28（6.52） | 0（0.00） | 0（0.00） |
| I'm confident that this post will give the opportunities to meet cultivation objectives in: PRACTICAL EXPERIENCE (procedures and treatments of chest drains, passing NG tubes, minor surgeries under local anesthetic, biopsies, fitting coils, injections, psychological therapies etc.) | 40（9.30） | 167（38.84） | 181（42.09） | 42（9.77） | 0（0.00） | 0（0.00） |
| I'm confident that this post will give the opportunities to meet cultivation objectives in: CLINICAL EXPERIENCE (examination skills, taking a history, deciding investigations and management, seeing a variety of patients in different settings etc.) | 68（15.81） | 167（38.84） | 195（45.35） | 0（0.00） | 0（0.00） | 0（0.00） |
| Supervised learning events (SLEs) have led to me reflecting on my clinical practice. | 83（19.30） | 264（61.40） | 83（19.30） | 0（0.00） | 0（0.00） | 0（0.00） |
| SLEs have helped me to identify areas in which I need to develop. | 69（16.05） | 264（61.40） | 97（22.56） | 0（0.00） | 0（0.00） | 0（0.00） |
| SLEs have enabled me to improve my practice. | 69（16.05） | 278（64.65） | 69（16.05） | 14（3.26） | 0（0.00） | 0（0.00） |
| How easy or difficult was it to get a suitable physician to complete an SLE with you? | 27（6.28） | 236（54.88） | 139（32.33） | 28（6.51） | 0（0.00） | 0（0.00） |
| I have access to a senior physician who is onsite at all times. | 27（6.28） | 250（58.14） | 153（35.58） | 0（0.00） | 0（0.00） | 0（0.00） |
| The senior physician onsite could advise on any clinical situation. | 41（9.53） | 250（58.14） | 111（25.81） | 28（6.51） | 0（0.00） | 0（0.00） |
| Handover and reporting systems | | | | | | |
| Handover arrangements always ensure continuity of care for patients between shifts. | 109（25.35） | 210（48.84） | 83（19.30） | 28（6.52） | 0（0.00） | 0（0.00） |
| Handover arrangements always ensure continuity of care for patients between departments. | 151（35.12） | 168（39.07） | 97（22.56） | 14（3.26） | 0（0.00） | 0（0.00） |
| Appropriate members of the multidisciplinary team are included in handover. | 110（25.58） | 167（38.84） | 111（25.81） | 28（6.51） | 14（3.26） | 0（0.00） |
| I have been made aware of how to report patient safety incidents and near misses. | 69（16.05） | 221（51.40） | 84（19.53） | 14（3.26） | 0（0.00） | 42（9.77） |
| There is a culture of proactively reporting concerns. | 69（16.05） | 152（35.35） | 167（38.84） | 0（0.00） | 0（0.00） | 42（9.77） |
| There is a culture of learning lessons from concerns raised. | 124（28.84） | 166（38.60） | 98（22.79） | 14（3.26） | 0（0.00） | 28（6.51） |
| I am confident that concerns are effectively dealt with. | 110（25.58） | 166（38.60） | 112（26.05） | 14（3.26） | 0（0.00） | 28（6.51） |
| When concerns are raised, the subsequent actions are fed back appropriately. | 96（22.33） | 180（41.86） | 98（22.79） | 28（6.51） | 0（0.00） | 28（6.51） |

Table 2 Clinical experience, clinical supervision, local teaching and overall satisfaction (n=430)

|  | Excellent, n (%) | Good, n (%) | Fair, n (%) | Poor, n (%) | Very poor, n (%) |
| --- | --- | --- | --- | --- | --- |
| How would you rate the quality of the local/departmental teaching? | 40（9.30） | 237（55.12） | 97（22.56） | 56（13.02） | 0（0.00） |
| How would you rate the quality of teaching (informal and bedside teaching as well as formal and organized sessions)? | 27（6.28） | 208（48.37） | 181（42.09） | 14（3.26） | 0（0.00） |
| How would you rate the quality of clinical supervision? | 14（3.26） | 138（32.09） | 223（51.86） | 55（12.79） | 0（0.00） |
| How would you rate the quality of clinical experience? | 54（12.56） | 84（19.53） | 237（55.12） | 55（12.79） | 0（0.00） |
| How would you rate the practical experience you were receiving? | 54（12.56） | 140（32.56） | 209（48.60） | 27（6.28） | 0（0.00） |

Table 3 Feedback frequency, clinical supervision and workload (n=430)

| Feedback frequency | Daily, n (%) | Weekly, n (%) | Monthly, n (%) | Less than 1x/month, n (%) | Never, n (%) |  |
| --- | --- | --- | --- | --- | --- | --- |
| How often (if at all) do you receive informal feedback from senior physicians about your performance? | 28（6.51） | 125（29.07） | 42（9.77） | 138（32.09） | 97（22.56） |  |
| clinical supervision and workload | Daily，n (%) | Weekly，n (%) | Monthly，n (%) | Less than 1x/month，n (%) | never，n (%) | NA， n (%) |
| How often (if ever) are you supervised by someone who you feel isn’t competent to do so? | 14（3.26） | 98（22.79） | 56（13.02） | 55（12.79） | 95（22.09） | 112（26.05） |
| How often (if ever) do you feel forced to cope with clinical problems beyond your competence or experience? | 14（3.26） | 98（22.79） | 42（9.77） | 164（38.14） | 42（9.77） | 70（16.28） |
| How often (if ever) are you expected to obtain consent for procedures where you feel you do not understand the proposed intervention and its risks? | 28（6.51） | 70（16.28） | 42（9.77） | 192（44.65） | 28（6.51） | 70（16.28） |
| Have you worked out of hours (this includes night shifts and weekends)? | 97（22.56） | 166（38.60） | 153（35.58） | 14（3.26） | 11（2.56） | 0（0.00） |
| How often (if at all) do your working pattern leave you feeling short of sleep when at work? | 153（35.58） | 166（38.60） | 111（25.81） | 0（0.00） | 0（0.00） | 0（0.00） |

NA = not applicable
